# Axillary lymph node metastasis in breast cancer: from historical axillary surgery to updated advances in the preoperative diagnosis and axillary management

**DOI:** 10.1186/s12893-025-02802-2

**Published:** 2025-02-27

**Authors:** Tong Wu, Qian Long, Liyun Zeng, Jinfeng Zhu, Hongyu Gao, Yueqiong Deng, Yi Han, Limeng Qu, Wenjun Yi

**Affiliations:** 1 Department of General Surgery, The Second Xiangya Hospital, Central South University, Changsha, Hunan 410011 China; 2 Clinical Research Center for Breast Disease in Hunan Province, Changsha, 410011 China

**Keywords:** Breast cancer, Axillary surgery, Axillary lymph node metastasis, Diagnosis, Treatment

## Abstract

Axillary lymph node status, which was routinely assessed by axillary lymph node dissection (ALND) until the 1990s, is a crucial factor in determining the stage, prognosis, and therapeutic strategy used for breast cancer patients. Axillary surgery for breast cancer patients has evolved from ALND to minimally invasive approaches. Over the decades, the application of noninvasive imaging techniques, machine learning approaches and emerging clinical prediction models for the detection of axillary lymph node metastasis greatly improves clinical diagnostic efficacy and provides optimal surgical selection. In this work, we summarize the historical axillary surgery and updated perspectives of axillary management for breast cancer patients.

## Introduction

Breast cancer represents the most frequently diagnosed malignancy among women worldwide [1]. The status of the axillary lymph nodes (ALN) serves as a critical prognostic factor in breast cancer and strongly influences the surgical approach and therapeutic options [2, 3]. Traditionally, ALN status is determined by axillary lymph node dissection (ALND), which may cause a large incision and complications [4–6]. Over the years, axillary management in early breast cancer has changed from ALND to sentinel lymph node biopsy (SLNB), which has a reduced incidence of complications [7]. Several clinical trials have concluded that in early-stage breast cancer patients, SLNB could be an alternative to ALND without significantly impacting locoregional recurrence or long-term survival [8–10]. In addition, for patients with no initial node involvement, negative sentinel lymph node (SLN) after neoadjuvant chemotherapy (NAC) allows to safely avoid an ALND [11, 12].

Imaging techniques have been extensively employed in the diagnosis of ALN metastasis in patients with breast cancer [13–15]. Ultrasound (US), computed tomography (CT), positron emission tomography/computed tomography (PET/CT), and magnetic resonance imaging (MRI) are the main options for diagnosis [16–19]. Ultrasound has long been routinely applied to assess ALN status preoperatively; however, it has a sensitivity and specificity of 26.4–92.0% and 55.6–98.1%, respectively [14, 20]. CT and MRI are characterized by high spatial resolution and low interobserver variability, improving the diagnostic performance for ALN metastasis [21–23]. PET/CT has been utilized to evaluate the staging of breast cancer and detect ALN metastasis but may cause unnecessary exposure to ionizing radiation [24]. Artificial intelligence (AI) has achieved remarkable success in medical applications, especially in disease diagnosis and treatment response based on imaging methods [25–28]. Machine learning, which was proposed in the 1950s, is the core of AI and has attracted much interest in the diagnosis of ALN metastasis in breast cancer patients in recent years [29, 30]. Imaging-based machine learning methods include two main approaches: radiomics and deep learning [31, 32]. In addition, there are various emerging clinical prediction models for detecting ALN metastasis as noninvasive tools for providing additional information for clinical decision-making [33–35].

**Fig. 1:**
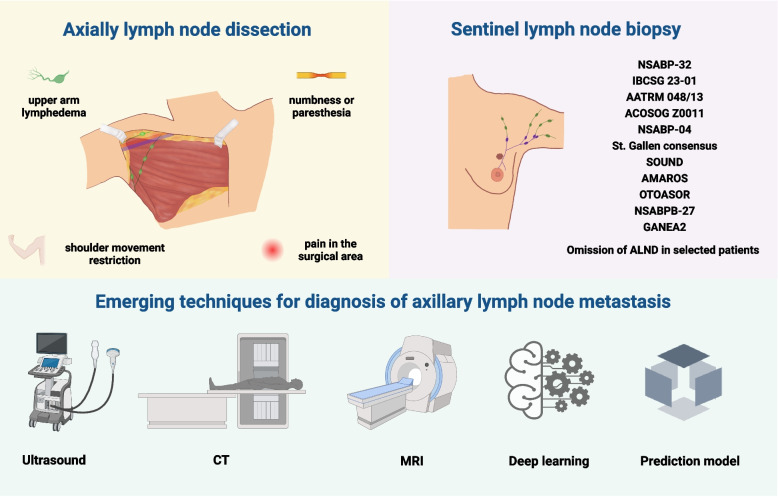
History of axillary surgery and emerging techniques for the detection of axillary lymph node metastasis. Axillary lymph node dissection (ALND) was performed as a standard method for determining axillary lymph node (ALN) status breast cancer patients until the 1990s, which may cause inevitable complications, such as upper arm lymphedema, shoulder movement restriction, numbness or paresthesia and pain syndrome in the surgical area, seriously affecting the life quality of breast cancer patients. Over the decades, increasing evidence has shown that sentinel lymph node biopsy (SLNB) could be an alternative option for ALND in selected patients, with no significant influence on axillary recurrence or long-term survival. To improve the diagnostic performance of metastatic axillary lymph nodes and reduce postoperative complications, various emerging techniques have been used as noninvasive approaches preoperatively. Imaging methods including ultrasound, computed tomography, magnetic resonance imaging, have long been the main options for diagnosis. Recently, machine learning approaches, which can automatically classify metastatic ALNs, have shown promise in the diagnosis of ALN metastasis. Clinical prediction models that combine imaging features or biomarkers and clinical factors also provide additional information for clinical decision-making. ALND, axillary lymph node dissection; CT, computed tomography; MRI, magnetic resonance imaging. Created with BioRender.com

In the current review, we summarize the development of axillary surgery and novel techniques in the preoperative diagnosis of ALN metastasis in patients with breast cancer. In addition, we introduce updated perspectives of treatment strategies for ALN metastasis in breast cancer (Fig. 1).

### Historical axillary surgery of breast cancer

**Fig. 2:**
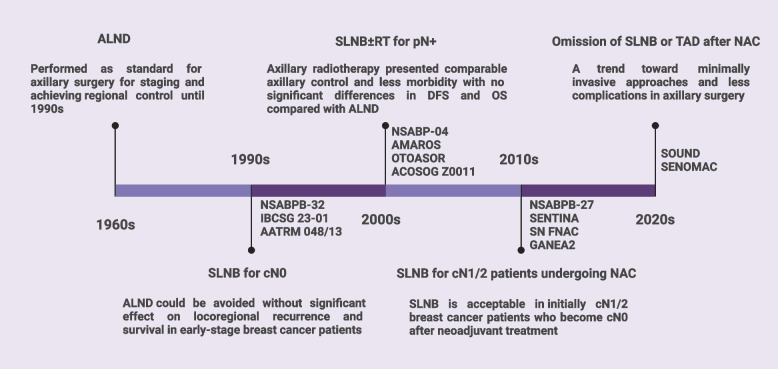
Clinical trials of axillary management in patients with breast cancer. Axillary lymph node status was routinely assessed by axillary lymph node dissection (ALND), which can provide information for staging and achieving regional control. Several clinical trials have been performed to determine whether ALND can be safely omitted and whether sentinel lymph node biopsy (SLNB) may serve as an alternative for ALND. For early-stage breast cancer patients, ALND could be avoided without significantly affecting locoregional recurrence or long-term survival. For T1-2 breast cancer patients with no palpable lymphadenopathy and a positive sentinel lymph node, axillary radiotherapy presents comparable axillary control and less morbidity. For patients who receive neoadjuvant chemotherapy (NAC) before axillary surgery, SLNB is acceptable in initially cN1/2 patients who become cN0 after NAC. There is a growing trend toward minimally invasive approaches and fewer postoperative complications in axillary surgery. ALND, axillary lymph node dissection; SLNB, sentinel lymph node biopsy; RT, radiotherapy; pN + , pathologically node-positive; DFS, disease-free survival; OS, overall survival; TAD, targeted axillary dissection; cN0, clinically node-negative; cN1/2, clinically node-positive; NAC, neoadjuvant chemotherapy. Created with BioRender.com

Axillary surgery for breast cancer has evolved characterized by gradual surgical de-escalation over the past 50 years, aiming to reduce postoperative morbidities and improve life quality of breast cancer patients [36–38]. Management of the axilla has progressed from ALND toward less invasive treatment with the use of SLNB [39]. There is a trend toward omitting SLNB or implementing targeted axillary dissection in the future [40] (Fig. 2).

ALND was performed as the standard for axillary surgery in breast cancer patients until the 1990s and was considered necessary for staging and achieving regional control in the long term [38, 41, 42]. However, ALND may cause various postoperative complications, including upper arm lymphedema, shoulder limitation, numbness, and pain syndrome in the surgical area, therefore prompting efforts toward surgical de-escalation of axillary surgery [43–45]. Several clinical trials have been conducted to assess the potential of SLNB as a viable alternative to ALND in selected patient populations (Table 1). The National Surgical Adjuvant Breast and Bowel Project (NSABP) trial B-32 randomized 5611 women with clinically negative lymph nodes into two groups: one group underwent SLN resection in conjunction with ALND (group 1), while the other group received SLN resection alone, with ALND performed only in cases where positive SLNs were identified (group 2) [8]. Overall survival (OS), disease-free survival (DFS), and the risk of recurrence were not significantly different between two groups [8]. The International Breast Cancer Study Group (IBCSG) 23–01 randomized controlled trial was conducted to compare the DFS and long-term surgical complications between the axillary dissection group and the no axillary dissection group of patients with the presence of one or more micrometastases measuring 2 mm or less in the SLNs. After a 9.7-year follow-up, two groups showed no differences in DFS or OS or in terms of complications [9, 46]. Consistent with IBCSG 23–01 trial, the AATRM 048/13 trial demonstrated that in early-stage breast cancer patients with micrometastatic SLNs, ALND could be avoided without significantly affecting locoregional recurrence or survival [47]. The American College of Surgeons Oncology Group Z0011 (ACOSOG Z0011) phase III randomized clinical trial was designed to assess the outcomes of patients subjected to breast-conserving surgery and SLN dissection alone and patients subjected to ALND. After a median follow-up of 10 years, the trial demonstrated that SLN dissection alone was noninferior to ALND when evaluating 10-year OS, DFS, and locoregional recurrence for patients with clinically node-negative breast cancer and a maximum of two positive SLNs who received breast-conserving surgery and adjuvant systemic therapy [10]. These trials illustrated that ALND is not justified for early-stage breast cancer patients who present with only one or two metastatic SLNs, thus reducing postoperative complications without significant effect on long-term survival outcomes. However, ALND remains the standard treatment in patients receiving breast-conserving surgery with a macroscopic lymph node or ≥ 3 positive SLNs and in patients undergoing mastectomy in cases of at least one metastatic SLN [10, 48, 49]. The SOUND (Sentinel Node vs Observation After Axillary Ultra-Sound) phase 3 randomized clinical trial was conducted to investigate the necessity of SLNB in patients with small breast cancer (equal to or smaller than 2 cm in diameter) and a negative preoperative ultrasonography result of axillary lymph nodes. The results demonstrated that the no axillary surgery group and SLNB group showed no significant difference in the incidence of locoregional relapses, distant metastases and deaths, and that the omission of axillary surgery was noninferior to SLNB in terms of distant disease-free survival at 5 years. In conclusion, patients with small breast cancer and negative axillary results on ultrasonography may be appropriately spared axillary surgery when the absence of pathological information does not influence the postoperative treatment strategy [50]. The SENOMAC (Sentinel Node Biopsy in Breast Cancer: Omission of Axillary Clearance After Macrometastases) trial [51] randomly assigned patients with clinically node-negative breast cancer into ALND group and SLNB group. Patients were also treated with adjuvant treatment and radiation therapy according to national guidelines. In patients with clinically node-negative breast cancer who had sentinel-node macrometastases and predominantly received nodal radiation therapy, omitting ALND was found to be noninferior to the more extensive surgical approach.

**Table 1:** Clinical trials for axillary management in breast cancer

Study	Design	Conclusion
NSABP (National Surgical Adjuvant Breast and Bowel Project) B-32 trial [8]	Group 1: SLNB and ALND Group 2: SLNB alone with ALND only if SLNs were positive	OS, DFS, and the risk of recurrence had no statistically significant differences between two groups
IBCSG (International Breast Cancer Study Group) 23–01 trial [46]	Group 1: no axillary dissection Group 2: axillary dissection	The group without axillary dissection showed no differences in DFS or OS, and less postoperative complications compared with the axillary dissection group
AATRM 048/13 trial [47]	Control arm: complete ALND Experimental arm: clinical follow-up	In early-stage breast cancer patients with micrometastatic SLNs, ALND could be avoided without significant effect on locoregional recurrence and survival
ACOSOG (American College of Surgeons Oncology Group) Z0011 trial [10]	Group 1: SLNB alone Group 2: ALND	SLNB alone was noninferior to ALND in terms of 10-year OS, DFS and locoregional recurrence for patients with cN0 breast cancer and no more than two positive SLNs
SOUND (Sentinel Node vs Observation After Axillary Ultra-Sound) trial [50]	Group 1: SLNB group Group 2: no axillary surgery group	Patients with small breast cancer and negative axillary results on ultrasonography can be safely spared any axillary surgery
SENOMAC (Sentinel Node Biopsy in Breast Cancer: Omission of Axillary Clearance After Macrometastases) trial [51]	Group 1: ALND Group 2: SLNB only	In patients with clinically node-negative breast cancer who had sentinel-node macrometastases and predominantly received nodal radiation therapy, omitting ALND was found to be noninferior to the more extensive surgical approach
NSABP-04 trial [52]	Patients with clinically negative axillary nodes: radical mastectomy, total mastectomy with postoperative irradiation, or total mastectomy with axillary dissection Patients with clinically positive axillary nodes: radical mastectomy, or total mastectomy with postoperative irradiation	There showed no significant differences in cases of axillary recurrence or survival among patients in all treatment groups with either clinically negative or positive axillary nodes
AMAROS (After Mapping of the Axilla: Radiotherapy Or Surgery) trial [53]	Group 1: ALND Group 2: axillary radiotherapy	In T1-2 breast cancer patients with no palpable lymphadenopathy and a positive SLN, axillary radiotherapy presented comparable axillary control and a lower risk of morbidity with no significant differences in DFS and OS compared with ALND
OTOASOR (Optimal Treatment Of the Axilla—Surgery Or Radiotherapy) trial [54]	Standard treatment: completion of standard treatment Investigational treatment: regional nodal irradiation	Axillary nodal irradiation could be an alternative therapy for ALND in selected patients with early-stage breast cancer (cN0) and low sentinel lymph node burden (pN1)
NSABPB-27 [55]	Group 1: SLNB and ALND Group 2: SLNB alone	SLNB may be an applicable option as an alternative for ALND in patients who have underwent NAC
European Institute of Oncology [11]	Group 1: initially cN0 patients Group 2: initially cN1/2 patients	SLNB is acceptable in initially cN1/2 breast cancer patients who become cN0 after NAC
SENTINA(SENTinel NeoAdjuvant) trial [56]	Arm A: initially cN0 patients treated with SLNB before NAC Arm B: pN1 patients treated with SLNB after NAC Arm C: ycN0 patients treated with SLNB and ALND Arm D: ycN1 patients treated with ALND without SLNB	SLNB is considered a reliable diagnostic technique before NAC. After systemic treatment or early SLNB, the procedure exhibits a lower detection rate and a higher false negative rate compared with SLNB done before NAC
SN FNAC study [57]	patients with biopsy-proven node-positive breast cancer (T0-3, N1-2) underwent SLNB and ALND	A low FNR of SLNB after NAC in biopsy-proven node-positive breast cancer can be achieved with the use of IHC for sentinel node evaluation
GANEA2 (Ganglion sentinel apres chimiotherapie NEoAdjuvante) trial [12]	Group 1: cN0 group Group 2: pN1 group	No initial node involvement and a negative SLN post NAC allow to safely spare an unnecessary ALND

*SLNB* sentinel lymph node biopsy, *ALND* axillary lymph node dissection, *SLN* sentinel lymph node, *OS* overall survival, *DFS* disease-free survival, *cN0* clinically node-negative, *pN1* pathologically node-positive, *NAC* neoadjuvant chemotherapy, *ycN0* clinically node-negative after therapy, *ycN1* clinically node-positive after therapy, *IHC* immunohistochemistry

Research efforts have been made to determine whether radiotherapy after mastectomy is as effective as ALND in terms of region-specific recurrence and long-term survival in early-stage breast cancer patients. The NSABP-04 trial, a randomized trial before the application of SLNB, assessed the efficacy of less invasive surgical approaches with or without radiation therapy in comparison to the Halsted radical mastectomy. Patients with clinically negative axillary lymph nodes received one of three surgical interventions: radical mastectomy, total mastectomy with regional irradiation, or total mastectomy with axillary dissection which was performed only if the lymph nodes tested positive. Patients with clinically positive axillary lymph nodes were subjected to either radical mastectomy or total mastectomy with postoperative irradiation. After a 25-year follow-up, the results showed no significant differences in cases of axillary recurrence or survival among patients in all treatment groups with either clinically negative or positive axillary nodes [52]. The phase 3 AMAROS (After Mapping of the Axilla: Radiotherapy Or Surgery) trial demonstrated that, in T1-2 breast cancer patients with no palpable lymphadenopathy and a positive sentinel lymph node, axillary radiotherapy presented comparable axillary control and a lower risk of morbidity, with no significant differences in DFS or OS compared with ALND [53]. The OTOASOR trial (Optimal Treatment Of the Axilla—Surgery Or Radiotherapy) reported similar conclusions that axillary nodal irradiation could be an alternative therapy to ALND in selected patients with early-stage breast cancer (cN0, cT ≤ 3 cm) and low sentinel lymph node burden (pN1) [54].

It is of some concern whether SLNB alone without ALND is acceptable for initially clinically node-positive (cN1/2) patients who convert to clinically node-negative (cN0) after NAC but have residual disease on final pathology at time of surgery. Many studies have been performed to assess the feasibility and efficacy of SLNB alone after NAC as an alternative to ALND. The NSABP B-27 trial introduced the first piece of evidence that SLNB could be an applicable option as an alternative to ALND in patients who have undergone NAC [55, 58, 59]. A retrospective study conducted by the European Institute of Oncology assessed 396 patients with cT1-4 and cN0/1/2 breast cancer who converted to or remained cN0 after NAC and received SLNB if at least one sentinel node was found. After a 61-month median follow-up, the OS in the whole cohort (90.7%), in initially cN0 patients (93.3%), and in initially cN1/2 patients (86.3%) showed no significant difference (*p* = 0.12). These findings lead to the conclusion that SLNB is acceptable in initially cN1/2 breast cancer patients who become cN0 after NAC [11]. In the SENTINA (SENTinel NeoAdjuvant) trial, patients with initially cN1/2 who converted cN0 after NAC underwent SLNB and ALND. Lymph node involvement was limited to the SLNs in 131(58%) of 226 patients. Besides, there was a significant association between the number of resected SLNs and the false-negative rate (FNR) [56]. The SN FNAC study [57] assessed the accuracy of SLNB after NAC in patients with biopsy-proven node-positive breast cancer. The mandatory implementation of immunohistochemistry for the SLN after NAC achieved a reduced FNR of SLNB. The GANEA2 (Ganglion sentinel apres chimiotherapie NEoAdjuvante) trial enrolled 957 patients treated with neoadjuvant therapy. Patients were randomly divided into the cN0 group and pN1 group based on lymph node involvement proven cytologically before NAC. After NAC, patients in the cN0 group received SLNB with ALND only in terms of sentinel node involvement, and patients in the pN1 group received SLNB and ALND. The results suggested that no initial node involvement or a negative SLN post-NAC allowed to safely spare an unnecessary ALND [12]. The 2017 St. Gallen consensus conference recommended that SLNB would be adequate if there are at least three or more negative sentinel nodes, while ALND is still needed in patients with clinically positive axilla or macrometastases in the SLN after neoadjuvant therapy [60]. ALND is the standard procedure for patient with pathologically node-positive disease detected after NAC [36]. The ongoing prospective Alliance A011202 trial (NCT01901094) was conducted to address the necessity of ALND in this patient population, comparing ALND with axillary radiotherapy in terms of extended regional nodal irradiation [61, 62]. Recruitment for the trial is nearing completion, and the analysis of the primary endpoint is anticipated to occur in the coming years. Until then, the de-escalation of ALND could be definitively addressed for the majority of these patients [63].

### Imaging-based approaches for the prediction and diagnosis of axillary lymph node metastasis

Preoperative diagnosis of metastatic ALNs helps to personalize surgical plans and achieve de-escalation of surgery. In recent years, the application of imaging methods for the diagnosis of ALN metastasis has become increasingly mature, including ultrasound (US), computed tomography (CT), positron emission tomography/computed tomography (PET/CT), and magnetic resonance imaging (MRI).

#### Ultrasound

Ultrasound has long been routinely applied in the preoperative evaluation of ALN status as a noninvasive technique in breast cancer patients [64, 65]. Metastatic lymph nodes have typical characteristics and provide critical information for axillary surgery [66]. Several studies have been conducted to evaluate the accuracy and efficacy of conventional gray-scale ultrasound in the diagnosis of ALN metastasis. A retrospective study that enrolled 162 patients with triple-negative breast cancer (TNBC) reported that the blood flow grade and the long-to-short axis (L/S) ratio of ALNs were independent predicting factors of metastatic lymph nodes, with area under the curve (AUC) of 0.6329 and 0.6498, respectively [66].

Contrast-enhanced ultrasound (CEUS) can show more blood flow and reveal tumor perfusion characteristics, which is reportedly better than conventional ultrasound for discriminating between benign and malignant tumors [67–69]. A meta-analysis performed by Liu et al. [70] investigated the predictive performance of CEUS in identifying metastatic SLNs in 12 studies of 1525 patients. The sensitivity and specificity yielded 0.91 and 0.86, respectively, and the AUC achieved 0.95. The findings concluded that CEUS served as a reliable imaging method for the diagnosis of SLN metastasis and stage management of breast cancer. Ultrasound elastography (UE) is a vital ultrasound imaging technique that can provide additional prognostic information along with conventional US [65]. UE utilizes the concept that cancer tissues are often stiffer than normal breast tissues to discriminate between malignant and benign breast lesions [71]. Xu et al. [71] evaluated the characteristics of 97 ALNs by both conventional gray-scale ultrasound and modified real-time elastography to diagnose metastatic ALNs. The results showed that UE had a better specificity compared with gray-scale ultrasound. Besides, the diagnostic efficacy of a combined evaluation approach was superior to that of either gray-scale ultrasound or UE alone, suggesting that UE may serve as a supplementary technique in addition to conventional ultrasound in the assessment of ALN metastasis. The combined application of two or more ultrasound examination techniques may help to improve diagnostic efficiency. Li et al. [72] conducted a network meta-analysis to investigate the prognostic performance of US, UE, CEUS, US + UE, and US + CEUS for ALN metastasis. Among the five groups, US + CEUS ranked first in sensitivity, specificity, and accuracy, indicating that US + CEUS may perform better in assessing ALN status in breast cancer patients.

#### CT and PET/CT

Compared with ultrasound, CT presents higher spatial and density resolution, which depicts the changes between tumor lesions and the surroundings [21]. Functional imaging modalities such as positron emission tomography (PET) can provide metabolic information, which can be valuable in detecting malignant lymph nodes [73, 74].

CT scans provide information about the size, location, and appearance of lymph nodes in the axilla and help to determine enlarged or suspicious lymph nodes [75, 76]. A study performed by KUTOMI et al. [77] analyzed the preoperative contrast CT images of 75 patients to evaluate whether CT could be used as a valuable modality for the diagnosis of ALN metastasis. The shape of lymph nodes was categorized into three distinct classifications: fat, clear and obscure types. Notably, clear-type lymph nodes emerged as a significant independent indicator of ALN metastasis. Chen et al. [78] conducted a study to evaluate the diagnostic efficacy of multidetector-row computed tomography (MDCT) in identifying metastatic lymph nodes. The results indicated that a cortical thickness exceeding 3 mm and the presence of a nonfatty hilum served as independent prognostic factors for ALN metastasis. The MDCT images exhibited excellent performance in metastasis prediction, achieving a sensitivity of 85.3%, specificity of 87.4%, and AUC of 0.893.

PET/CT plays a complementary role alongside other imaging modalities in the diagnosis and staging of ALN metastasis in patients with breast cancer [74]. It provides valuable information about metabolic activity, aiding in the detection of small metastatic deposits that may not be evident on anatomical imaging alone [79]. The applications of [18] [F]-fluorodeoxyglucose (^18^F-FDG) PET/CT for the assessment of ALN status have been the subject of investigation. A meta-analysis was conducted to assess the predictive efficacy of [18] F-FDG PET/CT in diagnosing ALN metastasis, revealing a specificity of 94% [80]. Davidson et al. [74] investigated the association between FDG uptake and ALN metastasis. A total of 81.8% of the diagnosed patients had localized uptake of FDG corresponding to malignant lesions, and 6.5% of the diagnosed patients had no FDG uptake. The FDG avidity was strongly associated with tumor size, clinical stage, and biological type. The results suggested that FDG PET/CT may be utilized as a staging technique for predicting ALN metastasis only in patients identified as having a high risk of regional metastasis.

#### MRI

MRI serves as a complemental tool in the diagnosis of ALN metastasis in breast cancer [81]. A previous study explored the predictive value of MRI for ALN metastasis. The sensitivity, specificity, and accuracy yielded over 90% and AUC was greater than 0.9 [23]. Zhou et al. [81] conducted a meta-analysis to evaluate the diagnostic performance of MRI for ALN metastasis with 26 studies included. With a sensitivity, specificity, and AUC of 0.77, 0.90, and 0.93, respectively, it can be concluded that MRI is an effective method for the differentiation of metastatic lymph nodes, contributing to decision-making for axillary surgical management. The integration of MRI and PET/CT has the potential to improve the predictive accuracy regarding metastatic ALNs. Sae-Lim et al. assessed the diagnostic efficacy of MRI, PET/CT, and their combined application in the identification of ALN metastasis. The results concluded that the synergistic use of MRI and PET/CT exhibited high predictive values for determining low-burden (≤ 2 positive nodes) ALN metastasis in patients with operable breast cancer, which may facilitate the de-escalation of axillary surgery [82].

### Application of machine learning in metastatic lymph node diagnosis

Although imaging methods have been employed for the detection of ALN metastasis, there still remain several notable problems, including a high FNR, the subjectivity of the radiologist, and the inability to automatically classify the metastatic ALN [83–85]. There is an increasing need to combine valuable techniques to classify metastatic ALNs automatically and enhance the diagnostic capability of ALN metastasis. Artificial intelligence has shown substantial promise in medical applications, especially machine learning for image-based disease diagnosis [86–89]. Radiomics and deep learning are two commonly used machine learning approaches and have been demonstrated to be promising in the prediction of ALN metastasis [32]. Among the deep learning approaches used for metastatic ALN diagnosis, the convolutional neural network (CNN) is the most commonly applied [31]. Radiomics refers to the process of extracting a substantial quantity of imaging features such as texture, shape, size and intensity by high-throughput methods, thereby transforming medical images into high-dimensional data that can be analyzed [90, 91]. A CNN is composed of a series of convolutional layers that are adept at acquiring a compact hierarchical representation of the input data, which is tailored to effectively address the specific task [31].

Radiomics based on imaging methodologies have shown excellent performance in predicting ALN metastasis [75, 92]. Yang et al. [75] constructed a radiomics model employing a support vector machine algorithm, utilizing CECT images to detect ALN metastasis, which extracted 396 features from CECT images of 825 ALNs. The radiomics model yielded accuracies of 88.5% and 89.1% and AUCs of 0.94 and 0.92 in the testing and validation cohorts, respectively. Liu et al. [93] developed a radiomic signature from dynamic contrast-enhanced magnetic resonance imaging (DCE-MRI) images, which extracted 590 radiomic features of the primary tumor from intratumoral and peritumoral regions, with the analysis incorporating clinicopathologic characteristics, either in combination or isolation. The model using radiomic features alone achieved an AUC of 0.806. Combining DCE-MRI radiomic features with clinicopathologic factors, the signature yielded a higher AUC of 0.869. A retrospective study by Yu et al. [94] developed DCE-MRI radiomic signatures for the detection of ALN metastasis and evaluation of DFS in early-stage breast cancer patients. A total of 1214 patients were divided into development and validation cohorts to evaluate the diagnostic efficacy of the radiomic signature, the clinical signature, and the clinical-radiomic nomogram for identifying ALN metastasis. The clinical-radiomic nomogram yielded AUCs of 0.92 and 0.90 in the development cohort and validation cohort, respectively, outperforming both the radiomic signature, which yielded AUCs of 0.88 and 0.85, and the clinical signature, which produced AUCs of 0.77 and 0.71. Furthermore, the clinical-radiomic nomogram also exhibited superior performance in assessing 3-year DFS, achieving AUCs of 0.89 and 0.90, respectively. The results concluded that the clinical-radiomic nomogram performed well in the detection of ALN metastasis and provided personalized decisions related to therapeutic selection. In a retrospective study, Song et al. [95] constructed and validated a radiomics nomogram utilizing DCE-MRI images and clinical characteristics for the preoperative diagnosis of ALN metastasis. The radiomics signature yielded AUCs of 0.847 and 0.805 in the training and validation cohorts, respectively. In contrast, the clinical model, which incorporated histological grade, multifocality, and ALN status, achieved AUC values of 0.732 and 0.738, respectively. The combined model exhibired superior performance, with AUCs of 0.907 and 0.874, respectively. This radiomics nomogram provided a practical noninvasive method for the prediction and diagnosis of ALN metastasis.

Deep learning approaches, especially CNNs, have made significant contributions to image analysis and disease diagnosis [31]. Unlike radiomics which involves a manual step in feature extraction, CNNs can automatically classify and segment medical images to identify regions of interest and extract specific features from large amounts of quantitative data [96]. CNNs have been used to classify ALN metastasis based on imaging methods. Ren et al. [97] developed a CNN predictive model that involved 66 abnormal nodes and 193 normal nodes based on MRI scans to detect ALN metastasis. The CNN model achieved a specificity of 79.3%, sensitivity of 92.1%, accuracy of 84.8%, and AUC of 0.91. Chen et al. [98] constructed a CNN-based model aimed at predicting SLN and non-SLN metastasis using DCE-MRI images preoperatively. For SLN prediction, the CNN-based model achieved AUC values of 0.899, 0.885, and 0.768 in the validation set, test set 1, and test set 2, respectively. For non-SLN prediction, the AUCs yielded 0.800, 0.763, and 0.728, respectively. The results concluded that the CNN model based on DCE-MRI images may serve as a noninvasive approach for predicting the ALN status, thereby facilitating personalized axillary treatment in breast cancer patients. The diagnostic performance of the models mentioned above is summarized in Table 2.

**Table 2:** Machine learning models for the diagnosis of axillary lymph node metastasis

References	Model	Imaging method	Sensitivity	Specificity	Accuracy	AUC
Yang et al. [75]	radiomics model	CECT	0.882 in the testing cohort 0.824 in the validation cohort	0.887 in the testing cohort 0.963 in the validation cohort	0.885 in the testing cohort 0.891 in the validation cohort	0.94 in the testing cohort 0.92 in the validation cohort
Liu et al. [93]	radiomics model	DCE-MRI	0.901 in the training set 0.778 in the validation set	0.833 in the training set 0.861 in the validation set	0.896 in the training set 0.886 in the validation set	0.914 in the training set 0.869 in the validation set
Yu et al. [94]	clinical-radiomics nomogram	DCE-MRI	/	/	/	0.92 in the development cohort 0.90 in the validation cohort
Song et al. [95]	clinical-radiomics nomogram	DCE-MRI	0.821 in the training cohort 0.759 in the validation cohort	0.837 in the training cohort 0.845 in the validation cohort	/	0.907 in the training cohort 0.867 in the validation cohort
Ren et al. [97]	CNN model	MRI	0.921	0.793	0.848	0.91
Chen et al. [98]	CNN model	DCE-MRI	0.755	0.883	0.892	0.899

*AUC* area under the curve, *CECT* contrast-enhanced computed tomography, *DCE-MRI* dynamic contrast-enhanced magnetic resonance imaging

### Clinical prediction models for detection of axillary lymph node metastasis

Recent studies developing novel clinical prediction models that integrate imaging characteristics or biomarkers and clinical factors to evaluate the prediction of lymph node metastasis have achieved great success (Table 3).

**Table 3:** Clinical prediction models for the diagnosis of axillary lymph node metastasis

References	Model	Design	AUC
Shiino et al. [99]	serum miRNA-based prediction model	Combining serum miRNAs signature and clinicopathologic features	0.86
Tan et al. [34]	immune-related genes nomogram	Integrating the immune-based signature and ultrasound-based ALN status	0.87
Qu et al. [100﻿]	3D reconstruction system	Establishing a 2D formula, a sphericity formula, a decision tree model and a random forest model	0.844

*AUC* area under the curve, *ALN* axillary lymph node, *3D* three-dimensional, *2D* two-dimensional

Several biomarkers have been identified to detect ALN metastasis in breast cancer patients. Published studies have shown the predictive value of serum miRNAs for breast cancer [101–103]. Shiino et al. [99] developed a prediction model based on serum miRNAs and clinicopathologic factors for the evaluation of ALN status. Serum samples were subjected to analysis using miRNA microarray technology and were subsequently divided into a training set and a test set through random allocation. The results from the test set indicated a sensitivity of 0.88, a specificity of 0.69, an accuracy of 0.818, and an AUC of 0.86, demonstrating that the integration of serum miRNAs with clinicopathologic characteristics has the potential to function as a less invasive biomarker for the identification of ALN metastasis. Immune-related molecules have been involved in the carcinogenesis of breast cancer [104]. To assess the diagnostic performance of immune-related molecules for ALN metastasis, Tan et al. [34] developed an immune-related gene nomogram that integrated the immune-based signature and ultrasound features of ALNs in patients with TNBC. By analyzing RNA-Seq gene expression data of TNBC patients from The Cancer Genome Atlas dataset, a 5-gene signature associated with ALN metastasis was identified to distinguish patients with ALN metastasis (AUC, 0.80). The immune-related gene nomogram (AUC = 0.87), which incorporated the 5-gene signature with ultrasound-based ALN status, performed better than the immune-related gene signature alone (AUC = 0.80) or the model containing a single ultrasound feature of ALNs (AUC = 0.73). The immune-related gene nomogram proved to be a favorable biomarker for predicting ALN metastasis preoperatively and provided individual and noninvasive information for clinical decision-making.

The morphology of the lymph nodes can yield valuable insights for the diagnosis of ALN metastasis in patients with breast cancer, generally based on the L/S ratio. Three-dimensional (3D) imaging methods can better display spatial shape and morphological changes in suspected lymph nodes than current two-dimensional (2D) imaging methods, such as US, CT, MRI, and PET-CT. Qu et al. [100] established a 3D reconstruction system to assess lymph node status based on preoperative CT images from 43 breast cancer patients with lymph node pathological results. The research developed a 2D formula and a sphericity formula, and further established a decision tree model and a random forest model to detect lymph node metastasis in the training cohort. The random forest model achieved a sensitivity of 88.9%, a specificity of 80.0%, and an AUC of 0.844, indicating superior classification performance. In the validation cohort, the classification rates of the above four methods reached 69.8%, 86%, 88.4%, and 90.7%, respectively, which were significantly higher than those of ultrasound (38.5%) and CT (48.8%), suggesting that the 3D reconstruction system could better reflect morphological changes and had greater diagnostic efficiency.

### Future perspectives and conclusions

Axillary lymph node status influences the surgical approach, therapeutic options, and long-term survival of breast cancer patients [2]. In recent years, there have been great advancements and ongoing developments in axillary surgery and treatment of ALN metastasis for breast cancer.

Emerging evidence has shown that SLNB may safely serve as an alternative to ALND in selected patients [105]. In the near future, axillary surgery may evolve to less invasive approaches, such as targeted axillary dissection (TAD), which has been recommended by several guidelines, despite insufficient long-term follow-up regarding its oncologic outcomes [106, 107]. TAD has become increasingly popular for staging the axilla in patients with clinically node-positive who converted to cN0 after NAC, due to its lower FNR and minimal arm complications [108]. TAD is a more precise approach that aims to reduce the extent of lymph node removal while still providing essential data for staging and treatment planning [109, 110]. The procedure involves the removal of SLNs as well as the marked nodes which are first indicated with image-detectable markers [107, 111]. Compared to SLNB for cN1 patients after NAC, TAD reduces the FNR [112] and decreases the incidence of postoperative complications such as upper limb morbidity [113], thus helping to reduce postoperative pain and discomfort, and improve quality of life in breast cancer patients. In the study conducted by Laws et al., SLNB and TAD demonstrated equivalent rates of technical failures and low rates of axillary recurrence in cN1 patients after NAC [114]. In addition, it is worth noting that with the overall trend towards omitting nodal surgery entirely, the detection and diagnose of occult axillary metastases is of great importance, as it is may be associated with the recurrence risk and survival outcomes of breast cancer patients. Previous research has reported that occult metastases can be detected in 9% to 33% of patients who initially present with negative lymph nodes determined by conventional pathological examination of ALND specimens [115]. However, the clinical relevance of occult metastases remains controversial, as the correlation between occult metastases and survival outcomes is inconclusive in the published studies [116–119]. Research have shown that serial sectioning and immunohistochemistry staining could improve the detection rate of occult metastases [120]. The improvement of methodologies has the potential to significantly increase the detection rate of occult metastases, but the implications for long-term survival requires further follow-up [121]. When occult metastases are detected which are considered negative using conventional methodologies, treatment for these patient population may need to be extended, such as increasing adjuvant systemic therapy and axillary radiotherapy [121]. Due to the lack of standardization in pathological examination and therapy, the prognostic significance of occult metastases remains constrained, and long-term investigation and further study are needed.

Immunotherapy, as an emerging treatment approach, has gradually shown potential in the treatment of axillary lymph node metastasis in breast cancer. Tumor microenvironment (TME) is a critical factor influencing the progression and metastasis of breast cancer, which could be a therapeutic target focused on metastasis [122, 123]. Chemokines have been found to show a key role in cancer progression and metastasis [124, 125]. The research conducted by Qiu et al. [126] demonstrated a correlation between the expression levels of C–C motif chemokine ligand-5 (CCL5) and lymph node metastasis, as well as clinicopathological factors of breast cancer. CCL5 affects the prognosis and metastasis of breast cancer through C–C motif chemokine receptor 5 (CCR5)/Treg signaling pathway, which may serve as a potential therapeutic target for immunotherapy against breast cancer. Recently, programmed cell death 1 (PD-1) and programmed cell death ligand 1 (PD-L1) have become promising targets for the treatment of TNBC [127]. Anti-PD-L1/anti-PD-1 therapy has shown good efficacy for TNBC patients, and the expression of PD-L1 could be used as a biomarker for prediction of the responses to PD-1/PD-L1-blockade therapy in TNBC patients [128, 129]. Li et al. [130] found that the expression levels of PD-L1 were significantly elevated in metastatic lymph nodes compared to those in the primary tumors. PD-L1 expression showed a positive association with both histological grade and the score of tumor-infiltrating lymphocytes (TIL). Furthermore, PD-L1 expression in the metastatic lymph nodes exhibited a significant correlation with increased rates of recurrence and distant metastasis. Moreover, the expression of PD-L1 in metastatic lymph nodes exhibited independent prognostic significance for DFS. The results revealed that PD-L1 status of metastatic lymph nodes could serve as a predictive marker for the response to PD-1/PD-L1-targeted therapies and help to select optimal therapy.

By combining multiple diagnostic methods, treatment for axillary lymph node metastasis has evolved toward individualization and precision, allowing physicians to more accurately select treatment options based on genotype, phenotype, and biomarkers to improve outcomes and reduce adverse effects [131, 132]. Besides, the approach of combination therapy, which encompasses the application of surgery, radiotherapy, chemotherapy, targeted therapy, and immunotherapy, has emerged as the predominant strategy for the treatment of axillary lymph node metastasis in breast cancer. This multifaceted treatment regimen has the potential to substantially enhance therapeutic efficacy and improve survival outcomes [133, 134].

In summary, axillary surgery for breast cancer patients has evolved to less invasive approaches, and the emergence of novel diagnostic techniques has greatly improved the detection rate of metastatic axillary lymph nodes, which enables more accurate localization of targeted lymph nodes and allows for personalized surgical selection. The treatment of axillary lymph node metastasis of breast cancer is evolving toward individualized, targeted, and comprehensive development. With the progress of scientific research and technology, it is expected that more innovative treatment strategies and new drugs will be applied in clinical practice in the future.

## Data Availability

No datasets were generated or analysed during the current study.

## Abbreviations

2D: Two-dimensional
3D: Three-dimensional
AI: Artificial intelligence
ALN: Axillary lymph node
ALND: Axillary lymph node dissection
AUC: Area under the curve
CCL5: C–C motif chemokine ligand-5
CCR5: C–C motif chemokine receptor 5
CEUS: Contrast-enhanced ultrasound
cN +: Clinically node-positive
cN0: Clinically node-negative
CNN: Convolutional neural network
CT: Computed tomography
DCE-MRI: Dynamic contrast-enhanced magnetic resonance imaging
DFS: Disease-free survival
FDG: Fluoro-D-glucose
FNR: False negative rate
L/S: Long-to-short axis
MDCT: Multidetector-row computed tomography
MRI: Magnetic resonance imaging
NAC: Neoadjuvant chemotherapy
OS: Overall survival
PD-1: Programmed cell death 1
PD-L1: Programmed cell death ligand 1
PET/CT: Positron emission tomography/computed tomography
pN1: Pathologically node-positive
SLN: Sentinel lymph node
SLNB: Sentinel lymph node biopsy
TAD: Targeted axillary dissection
TIL: Tumor-infiltrating lymphocytes
TME: Tumor microenvironment
TNBC: Triple-negative breast cancer
UE: Ultrasound elastography
US: Ultrasound
